# Assessing oral comprehension with an eye tracking based innovative device in critically ill patients and healthy volunteers: a cohort study: authors' response

**DOI:** 10.1186/s13054-023-04334-8

**Published:** 2023-02-01

**Authors:** Hélène Messet-Charrière, Stephan Ehrmann, Laetitia Bodet-Contentin

**Affiliations:** 1 CHRU Tours, Médecine Intensive Réanimation, Tours, France; 2 CHRU Tours, Médecine Intensive Réanimation, CIC INSERM 1415, CRICS-TriggerSep FCRIN research network, Tours, France; 3 INSERM, Centre d’étude des pathologies respiratoires, U1100, Université de Tours, Tours, France; 4 INSERM, SPHERE, UMR1246, Université de Tours et Nantes, Tours et Nantes, France

Dear Editors,

We thank Waydhas and colleagues for their interest and careful reading of our work [1, 2]. Addressing the questions raised will enable us to clarify the subject and further confirm the great potential of eye tracking technology for intensive care unit patients.

First, it was suggested that the performance of patients could be perceived as low, with a correct answer rate close to a theoretical guessing rate calculated by Waydhas. For technical reasons beyond the scope of this letter exchange, the correct answer rate one would expect by guessing is much lower than this calculation. Thus, the readers should not consider the numeric value as an indication that eye tracking would not be suited for critically ill patients. Actually, it is the opposite, our work validates the potential use of this technology in this setting.

Concerning gaze fixation time, whereas our group also performed similar studies on icons fixation for eye tracking-based electronic communication [3, 4], the setting and objectives of the present study (developing a diagnostic tool to quantify comprehension) were completely different.

We share the concern that the questions of the Montreal-Toulouse-86 test have little to do with the situation lived by patients which represents a bias inherent to using a test developed for another context. Hopefully, collaboration of teams with shared interest may enable to develop eye tracking fit tests specifically adapted to the intensive care unit setting.

The aim of our study was to assess the feasibility of an oral comprehension test using eye tracking technology with the ultimate goal to improve communication of the patient with the staff and family members. By early detection and precise quantification of comprehension difficulties, we envision to implement corrective measure and use appropriate personalized tools to communicate with patients.

We are strong believers of the importance of personal human interactions for communication in the intensive care unit and consider machines as a potential help but in no way a substitute. Waydhas and colleagues can be reassured on this point, and we strongly believe eye tracking technology has great potential to contribute to improved human communication leading towards a more humanized intensive care unit.

## Data Availability

Not applicable.
